# Comprehensive Serology Based on a Peptide ELISA to Assess the Prevalence of Closely Related Equine Herpesviruses in Zoo and Wild Animals

**DOI:** 10.1371/journal.pone.0138370

**Published:** 2015-09-17

**Authors:** Azza Abdelgawad, Robert Hermes, Armando Damiani, Benjamin Lamglait, Gábor Á. Czirják, Marion East, Ortwin Aschenborn, Christian Wenker, Samy Kasem, Nikolaus Osterrieder, Alex D. Greenwood

**Affiliations:** 1 Leibniz Institute for Zoo and Wildlife Research, Berlin, Germany; 2 Institut für Virologie, Freie Universität Berlin, Berlin, Germany; 3 Réserve Africaine de Sigean, 19 chemin du hameau du lac, Sigean, France; 4 Bwabwata Ecological Institute, Susuwe, Zambezi Region, Namibia; 5 Zoo Basel, Binningerstrasse 40, Basel, Switzerland; 6 Virology Department, Faculty of Veterinary Medicine, Kafrelsheikh University, Kafrelsheikh, Egypt; The University of Melbourne, AUSTRALIA

## Abstract

Equine herpesvirus type 1 (EHV-1) causes respiratory disorders and abortion in equids while EHV-1 regularly causes equine herpesvirus myeloencephalopathy (EHM), a stroke-like syndrome following endothelial cell infection in horses. Both EHV-1 and EHV-9 infections of non-definitive hosts often result in neuronal infection and high case fatality rates. Hence, EHV-1 and EHV-9 are somewhat unusual herpesviruses and lack strict host specificity, and the true extent of their host ranges have remained unclear. In order to determine the seroprevalence of EHV-1 and EHV-9, a sensitive and specific peptide-based ELISA was developed and applied to 428 sera from captive and wild animals representing 30 species in 12 families and five orders. Members of the *Equidae*, *Rhinocerotidae* and *Bovidae* were serologically positive for EHV-1 and EHV-9. The prevalence of EHV-1 in the sampled wild zebra populations was significantly higher than in zoos suggesting captivity may reduce exposure to EHV-1. Furthermore, the seroprevalence for EHV-1 was significantly higher than for EHV-9 in zebras. In contrast, EHV-9 antibody prevalence was high in captive and wild African rhinoceros species suggesting that they may serve as a reservoir or natural host for EHV-9. Thus, EHV-1 and EHV-9 have a broad host range favoring African herbivores and may have acquired novel natural hosts in ecosystems where wild equids are common and are in close contact with other perissodactyls.

## Introduction

The order Perissodactyla includes the three families *Equidae*, *Rhinocertidae*, and *Tapiridae*. In the *Equidae*, nine herpesviruses have been identified, six of which are allocated to the subfamily *Alphaherpesvirinae* and three in the subfamily *Gammaherpesvirinae*. EHV-1 is an alphaherpesvirus and arguably one of the most important equine pathogens with a worldwide distribution in domestic horses (*Equus ferus caballus*) in which it causes respiratory disease, abortion, neonatal death and neurological disorders [1]. Infections with EHV-1 or closely related viruses have been identified in other equids including zebras, domestic donkeys, and onagers [2–4]. Among non-equid perissodactyls, EHV-1 infection was reported in the Indian tapir (*Tapirus indicus)* and black rhinoceros *(Diceros bicornis)* [5, 6]. EHV-1 antibodies were detected with a prevalence of 8.8% in African white (*Ceratotherium simum*) and black (*Diceros bicornis*) rhinoceroses [5].

EHV-9, the most recently discovered equine alphaherpesvirus, was first described in captive Thomson’s gazelle (*Gazelle thomsoni*) in Japan that suffered from neurological symptoms and died [7], but was considered an accidental host [8–10]. Neither the natural host nor the complete host range of EHV-9 is known, but EHV-9 causes lethal disease in a number of different species under experimental conditions [3, 7, 10–13]. Both EHV-1 and/or EHV-9 have been shown to infect species in captivity other than their known natural hosts, resulting in disease and fatality in non-perissodactyla species such as polar bear *(Ursus maritimus)*, black bear *(U*. *americanus)*, llamas *(Lama glama)*, alpacas *(Vicugna pacos)*, blackbuck *(Antelopa cervicapra)*, Thomson’s gazelle (*Gazelle thomsoni*) and giraffe (*Giraffa camelopardalis*) [7, 12, 14–18]. A recombinant zebra-EHV-1/EHV-9 infection was reported in a polar bear and in Indian rhinoceros (*Rhinoceros unicornis*), in both cases resulting in severe and ultimately fatal neurological symptoms [19, 20]. The complete host range of EHV-1 and EHV-9 and whether there are differences in captivity that potentially promote cross-species transmission remains unknown.

Serological studies conducted on free-living zebra populations (*Equus burcelli*) have demonstrated the presence of antibodies against EHV-1 and EHV-4 in South Africa and against EHV-9 in Tanzania where zebras share water sources and grazing areas with Thomson’s gazelles and are thus frequently in close proximity to each other [3, 21, 22]. In Tanzania, the seroprevalence of EHV-1 and EHV-9 infections of 14% and 60%, respectively, was surveyed by serum neutralization test (SNT) [3]. However, equid alphaherpesviruses are very similar genetically and antigenetically; thus, SNT’s have poor discriminatory power for closely related viruses such as EHV-1, EHV-4 and EHV-9. Therefore, it is unlikely that SNT’s allow discrimination between EHV-1 or EHV-9 antibodies [7, 10].

To accurately distinguish between the different virus strains, a type-specific-gG-based enzyme-linked immunosorbent assay (ELISA) was developed for EHV-1- and EHV-4-specific antibodies [23]. ELISAs were also developed to discriminate between EHV-1 and EHV-4 using peptides [glycoprotein E (gE) for EHV-1 and glycoprotein G (gG) for EHV-4] [24, 25]. However, assays to discriminate antibodies against EHV-1 and EHV-9 have not been developed.

In this study we developed and applied a peptide-based ELISA to detect and differentiate between EHV-1- and EHV-9-specific antibodies in serum of different species. Seroprevalence for both viruses was determined from 428 serum samples collected from captive and wild animals. The objectives of the study were to determine the prevalence of EHV-1 and EHV-9 infections and specifically to determine the host range in the wild and to identify equid and non-equid reservoir species.

## Materials and Methods

### Serum samples

Sera were collected from non-vaccinated captive (n = 277 samples from 43 zoos) and free ranging species (n = 151 samples, Tanzania and Namibia) (Table 1). EHV-9 positive serum collected from an experimentally infected rabbit [26] was used as a positive control as no infected horse serum was available. An EHV-9 negative horse (umbilical cord blood serum) [24] sample was used as a negative control. An EHV-1-positive control serum collected at day 21 from an experimentally infected seronegative horse (horse a [24, 27]) and EHV-1-negative control (umbilical cord blood serum) collected from equine neonates immediately after birth [24] were used as controls for EHV-1 infection. Fetal calf serum was used as a negative control for SNT.

**Table 1:** List of animal species and number of serum samples used in this study.

	Zoo serum samples			
Order/*Family*/*species*/ *subspecies*		Number	Origin	Place of birth
**Perissodactyla**	**Odd-toed ungulates**			
***Equidae***			Captive	Captive
*Equus quagga antiquorum*	Damara zebra	6		
*Equus quagga boehmi*	Grant’s zebra	32		
*Equus quagga chapmani*	Chapman's zebra	1		
*Equus grevyi*	Grévy's zebra	17		
*Equus zebra hartmannae*	Hartmann's mountain zebra	33		
*Equus kiang*	Kiang	3		
*Equus ferus caballus*	Pony	4		
*Equus hemionus onager*	Onager	5		
*Equus africanus asinus*	Donkey	12		
*Equus africanus somalicus*	Somali wild ass	19		
***Rhinocerotidae***			Captive	Captive
*Ceratotherium simum*	White rhinoceros	64		26 captive
				36 wild
				2 unknown
*Diceros bicornis*	Black rhinoceros	6		Captive
**Artiodactyla**	**Even-toed ungulates**		Captive	Captive
***Bovidae***				
*Tragelaphus imberbis*	Lesser kudu	13		
*Hippotragus niger*	Sable antelope	12		
*Kobus megaceros*	Nile lechwe	7		
*Addax nasomaculatus*	Addax antelope	2		
*Taurotragus oryx*	Common eland	2		
*Ovibos moschatus*	Muskox	1		
***Giraffidae***				
*Giraffa camelopardalis*	Giraffe	10		
*Okapia johnstoni*	Okapi	8		
***Cervidae***				
*Alces alces alces*	Moose	1		
***Hippopotamidae***				
*Hippopotamus amphibious*	Hippopotamus	1		
**Carnivore**			Captive	Captive
***Ursidae***				
*Ursus maritimus*	Polar bear	7		
*Ursus thibetanus*	Asian black bear	8		
***Felidae***				
*Panthera leo krugeri*	White lion	1		
**Primates**			Captive	Unknown
***Cercopithecidae***				
*Papio hamadryas*	Hamadryas baboon	1		
*Erythrocebus patas*	Patas monkey	1		
	**Wild serum samples**			
**Perissodactyla**			Wild	Wild
***Equidae***				
*Equus quagga*	Plain zebra	41	40 Tanzania	
			1 Namibia	
***Rhinocerotidae***				
*Diceros bicornis*	Black rhinoceros	17	Namibia	
**Artiodactyla**			Namibia	Wild
***Bovidae***				
*Antidorcas marsupialis*	Springbok	20		
*Connochaetes gnou*	Black wildebeest	22		
**Carnivore**			Namibia	Wild
***Felidae***				
*Panthera leo*	Lion	17		
***Canidae***				
*Lycaon pictus*	African wild dog	6		
*Canis mesomelas*	Black-backed jackal	10		
***Hyaenidae***				
*Crocuta crocuta*	Spotted hyaena	8		
**Proboscidea**			Namibia	Wild
***Elephantidae***				
*Loxodonta africana*	African elephant	10		

### Ethics statement

The described research was approved by the Internal Ethics Committee of the Leibniz-institute for Zoo and Wildlife Research (IZW), Approval no. 2012-05-02.

### Serum neutralization test

Serum neutralization test (SNT) was performed as described in the OIE Manual of Diagnostic Tests and Vaccines for Terrestrial Animals with few modifications [3, 28]. Briefly, in 96-well micro plates, serial two fold dilutions of complement-inactivated serum samples at 56°C for 30 min were incubated with 100 plaque forming units (PFU)/100μl of EHV-1 or EHV-9 at 37°C. After 1 h incubation, 5×10^5^ equine dermal cells (ED) were added and incubated for 2 h at 37°C. The wells were overlaid with 1.6% (w/v) methylcellulose medium and incubated at 37°C. The reaction was stopped after 4 days with 3% (v/v) formalin and the plaques were stained with Giemsa. EHV-1-positive horse serum and fetal calf serum were included as positive and negative controls, respectively. In the case of EHV-9, positive rabbit serum and fetal calf serum were included as positive and negative controls, respectively. SN antibody titers were calculated by determining the highest serum dilution that completely protects the monolayers from infection (no CPE) in each well. Titers of ≥ 1:4 were considered as positive. Each test was validated with the positive and negative sera controls. Each serum sample was tested twice in duplicate independently.

### Peptides

Two sets of peptides, 18 amino acids (KQPQPRLRVKTPPPVTVP) for EHV-1_E (1E) and 15 amino acids (DSPPETPSPQENLND) for EHV-9_G (9G), that were biotinylated at the N-terminus and attached to two aminohexanoic acid hydrophobic spacers, to be able to attach to the streptavidin coated microtitre plates, were synthesized (Genscript, USA). The EHV-1_E peptide was used for descrimination between EHV-1 and EHV-4 as described previously [24]. EHV-9_G has shared 40% and 33% amino acid identity with gGs of EHV-1 horse strain (GenBank accession number: AET80923.1) and EHV-1 zebra strain (GenBank accession number: AII81244.1), respectively and was used to differenciate between EHV-1 and -9.

An EHV-1_G peptide (ESSLENQLTQEESNN), was tested with EHV-1 and EHV-9- positive and negative controls to confirm the specificity of the selected EHV-9_G peptide (GenBank accession number: AII81244.1).

### Peptide-based ELISA

The ELISA test was carried out on equids as described previously [24, 29] with few modifications. Briefly, 96-well plates (Sarstedt, Germany) were coated to 100 μl/well with 1μg/ml streptavidin dissolved in 50 nM carbonate-biocarbonate buffer (PH 9.6) overnight at 4°C. The wells were washed three times with PBS (PH 7.5) containing 0.1% (v/v) Tween 20 (PBST). After coating with 100 μl /well of the respective biotinylated peptide (2 μg/ml in 50 nM carbonate-biocarbonate buffer), the plates were incubated for 2 h at 37°C. Unoccupied sites were blocked by incubation for 1 h at 37°C with 1% (v/v) goat serum diluted in PBST. After washing, serum samples (100 μl/well) were added in dilution of 1:400 and incubated for 1 h at 37°C. Purified goat anti-horse IgG conjugated with horseradish peroxidase (1:20000; Dianova, Germany), and HRP-goat anti-rabbit IgG for the EHV-9 positive control (1:10000; cell signaling, Germany) was added to each well. After 1 h incubation and washing, the plates were developed with 100 μl/well TMB [3,3_,5,5_-tetramethylbenzidine; dissolved in 42 μg/ml citric acid, 0.01% (v/v) H_2_O_2_ (pH 3.95)]. The reaction was stopped after 10 min with 100 μl/ml of 1 M sulfuric acid and the plates were read at a wavelength of 450 nm on a spectrophotometer. Each serum sample was tested for significant EHV-1 and EHV-9 antibody titers at three independent times. Negative and positive serum controls were included in each plate.

Secondary antibody choice can be critical in serological analysis of different wildlife species [30]. For detection of EHV-1 and EHV-9 antibodies in different mammalian sera, the ELISA test was performed as described above except a 2% (w/v) albumin fraction was used as blocking buffer and protein G-peroxidase conjugate (1:10,000; Invitrogen, Germany) was used as a secondary antibody [30]. Protein G-peroxidase was shown to be capable of binding to the Fc region of immunoglobulin (IgG) without interfering with the antigen binding sites [30]. The protein G-peroxidase-dilution was optimized using 10 positive and 10 negative randomly selected zebra sera, based on our peptide-ELISA results. All negative samples yielded negative results with protein G-peroxidase and positive serum samples yielded the expected optical density (OD) values (S1 Fig). As controls, EHV-1 (wild zebra, PZ27) or EHV-9 (wild zebra, PZ34) positive zebra serum samples were used as a positive control. For testing carnivore serum samples, recombinant HRP-labeled protein A/G (1:10000; Thermo scientific, USA) was used [30].

### Assay sensitivity and specificity determination

The cutoff value is defined as the level of antibody activity which represents a minimum positive status for a tested animal. The method for determining a diagnostic cutoff is to test samples from known negative and positive populations [31]. In a previous study [24], the negative cutoff value for the EHV-1_E peptide was calculated based on data from 9 negative umbilical cord sera and 26 Icelandic horses sera, which were known to be immunologically naive to EHV-1. Due to the lack of specific pathogen free zebras and no EHV-1- or EHV-9-experimentally infected zebras, we used 27 zoo zebra sera (12 plains zebra, 9 Hartmann’s mountain zebra, and 6 Grevy’s zebra), which were free from detectable neutralizing antibodies against either EHV-1 or EHV-9 as tested by SNT (S1 Table), to calculate the negative cutoff value. A negative cutoff was calculated as the mean OD of zebra sera plus 2 SD and a positive cutoff was calculated as mean OD of this population plus 3 SD [24]. The cutoff values were applied to the non-equid samples as well because control serum and numbers of samples were insufficient to make an equivalent determination as with zebras and protein G peroxidase was shown not to interfere with the assay. The cutoff is conservative (mean OD plus or minus 2 to 3 SD) and it is more likely that some non-equids that were weakly positive were scored as negative than scoring animals as false positives.

### Statistical analysis

All statistical analyses and graphs were performed using GraphPad Prism version 5.0a software (GraphPad Software, San Diego, CA). Fisher tests were used to compare the frequencies or proportions of EHV-1 and -9 positivity. Differences were considered statistically significant when the *P* value was less than 0.05.

## Results

### Peptide ELISA sensitivity and specificity

**Fig 1:**
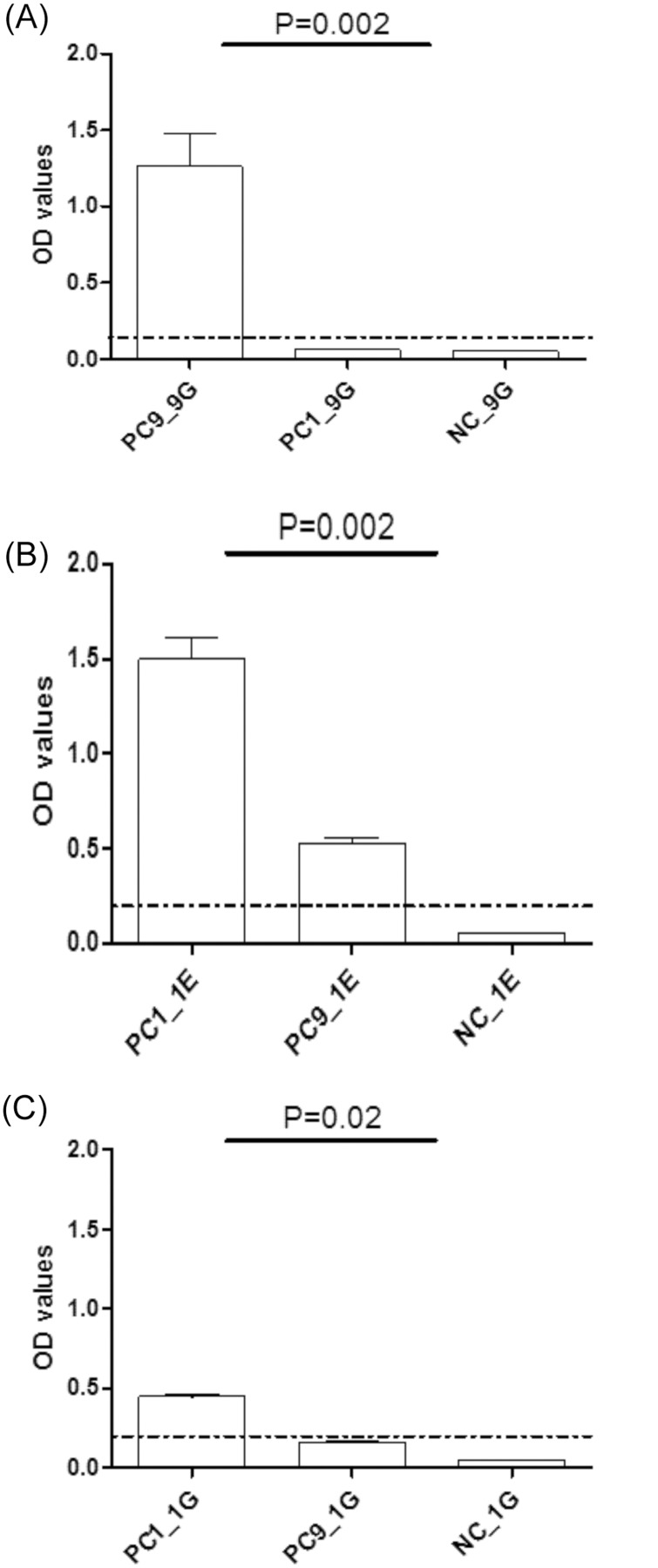
Reactivity and specificity of EHV peptides. Reactivity and specificity of (A) EHV-9_G (9G), (B) EHV-1_E (1E), and (C) EHV-1_G (1G) peptides using EHV-1 (PC1) (serum collected at day 21 from experimentally infected horse), EHV-9 (PC9) positive controls (rabbit serum) and NC (umbilical cord blood serum). The dashed line represents the negative cutoff value.

EHV-1 gE (EHV-1_E)- and EHV-9 gG (EHV-9_G)- peptides were used to differentiate between EHV-1- and EHV-9-specific antibodies. To test the reactivity and specificity of the selected EHV-9_G peptide, an EHV-9-positive rabbit serum and different positive and negative EHV-1 horse sera were used. The peptide produced high OD values (1.26) with an EHV-9-positive control serum and no reaction with EHV-1-positive (OD = 0.06) or EHV-9-negative (OD = 0.04) horse serum controls (Mann Whitney test, P = 0.002; Fig 1A).

The reactivity and specificity of the EHV-1_E peptide was previously determined [24]. The peptide produced high OD values (1.49) with an EHV-1-positive control, an OD value of 0.52 with the EHV-9-positive control and a low OD value (0.05) with EHV-1-negative controls. Although there was a significant difference between EHV-1- and EHV-9-positive controls (Mann Whitney test, P = 0.002; Fig 1B), this result did not unequivocally differentiate between EHV-1 and EHV-9 as the OD value of EHV-9-positive control was above the negative cutoff. An EHV-1_E peptide was used to detect EHV-1-positive antibodies, particularly in EHV-9-negative samples, and to exclude EHV-4 (a more distantly related virus to EHV-1 and EHV-9) infection [24].

To examine the specificity of the EHV-9_G peptide, EHV-1 and EHV-9 positive and negative controls were tested with a peptide with the EHV-1 gG sequence. The peptide produced OD values (0.4) with an EHV-1-positive control serum and OD = 0.15 with EHV-9-positive control and no reaction with EHV-9-negative (OD = 0.04) control (Mann Whitney test, P = 0.02; Fig 1C). Although it demonstrated a clear EHV-1 specificity, we did not rely on this peptide to discriminate the EHV-1 positive serum samples due to low reactivity with the EHV-1 positive control in comparison with the EHV-1_E peptide.

**Fig 2:**
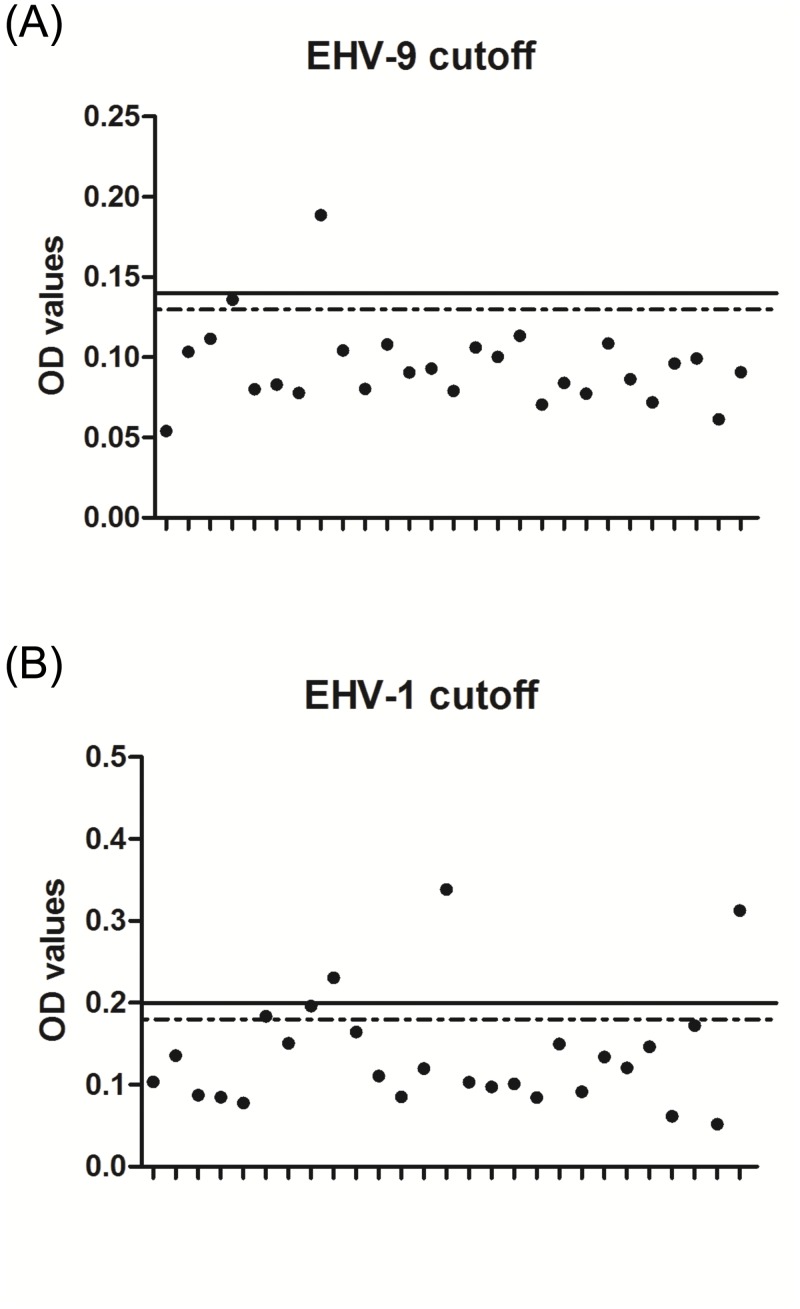
Determination of cutoff value. Twenty seven zebra serum samples were tested with either (A) EHV-9_G or EHV-1_E (B) peptides. OD values above the black line are considered positive, while OD values below the dashed line are considered negative. OD values between the lower and upper cut-offs were considered as questionable.

To determine the cutoff value, 27 zoo zebra sera were tested. The serum samples were considered EHV-1-positive when the OD value was higher than 0.2 and negative when the OD value was lower than 0.18. For EHV-9, serum samples were considered positive when OD values exceed 0.14 and negative when below 0.13. OD values between 0.18 and 0.2 in case of EHV-1 and between 0.14 and 0.13 in case of EHV-9 were considered questionable and may represent antibody levels that are either very low (at the detection limit) or non-specific cross reactions (Fig 2A and 2B). Three samples were tested positive for EHV-1, while one sample was tested positive for EHV-9.

### Detection of EHV-1 and EHV-9 neutralizing antibodies

None of the animals tested (Table 1), wild or captive, were noted to have displayed clinical symptoms including lesions associated with herpesvirus infection. Of 132 sera from equids in captivity, 60 and 59 were seropositive by SNT for EHV-1 and EHV-9, respectively (Table 2). In wild plains zebra serum samples (n = 41), 32 and 35 tested positive for EHV-1 and EHV-9, respectively (Table 2). The samples collected from captive kiangs, onagers or ponies (n = 12) were negative for antibodies against both viruses. Out of 64 captive white rhinoceros sera, 9 and 26 were positive by SNT for EHV-1 and EHV-9 antibodies, respectively. Captive black rhinoceros serum samples (n = 6) did not show neutralizing antibodies against EHV-1 or EHV-9. In wild black rhinoceros, one and two out of 17 animals were positive for EHV-1 and -9 antibodies, respectively (Table 2). None of the tested captive or wild non-equid sera tested positive for either virus, except for one captive antelope [lesser kudu (*Tragelaphus imberbis*)], which had a low antibody titer for EHV-9 (Table 2). In the current study, neutralizing antibodies for either EHV-1 or EHV-9 were not detected in any of the tested carnivore serum samples (captive: n = 16; wild: n = 41; Table 1).

**Table 2:** Equine herpesvirus-antibody positive rates in captive and wildlife sera using SNT.

		Number (%) of positive results	
*Family*/ animal	Total	EHV-1	EHV-9
Captive sera			
*Equidae*			
Plains zebra	39	24 (61.5%)	21 (53.8%)
Grevy's zebra	17	9 (52.9%)	9 (52.9%)
Hartmann's mountain zebra	33	10 (30.3%)	15 (45.4%)
Donkey	12	4 (33.3%)	3 (27.2%)
Somali wild ass	19	13 (68.4%)	11 (57.8%)
*Rhinocerotidae*			
White rhinoceros	64	9 (14.0%)	26(40.6%)
*Bovidae*	37	0 (0.0%)	1 (2.7%)
Wild sera			
*Equidae*			
Plains zebra	41	32 (78%)	35 (85.3%)
*Rhinocerotidae*			
Black rhinoceros	17	1 (5.8%)	2 (11.7%)

### Discrimination between EHV-1- and EHV-9- antibodies by peptide-based ELISA

**Fig 3:**
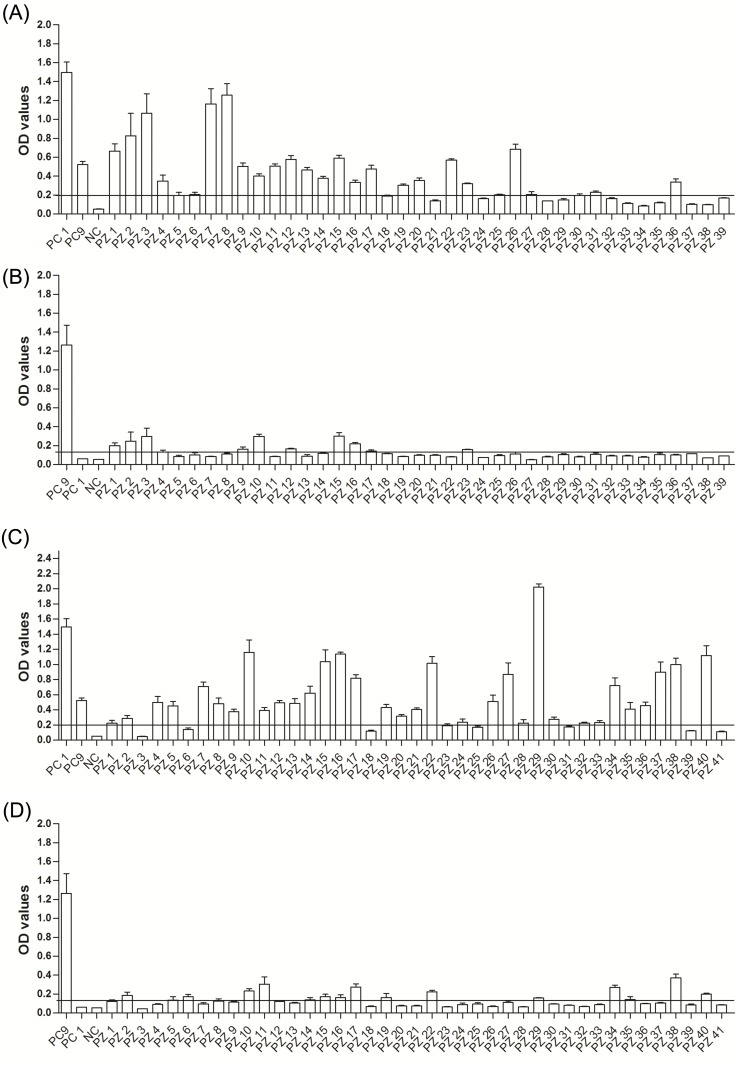
EHV antibodies in plains zebra. OD values of antibody responses tested by peptide-based ELISA in captive (A and B) and wild (C and D) plains zebra using EHV-1_E (A and C) and EHV-9_G (B and D) peptides. Black line: cutoff above which samples are considered positive. PC1 = EHV-1 positive control (serum collected at day 21 from experimentally infected horse), PC9 = EHV-9 positive control (serum collected from an experimentally infected rabbit), NC = negative control (umbilical cord blood serum), PZ = plains zebra.

**Fig 4:**
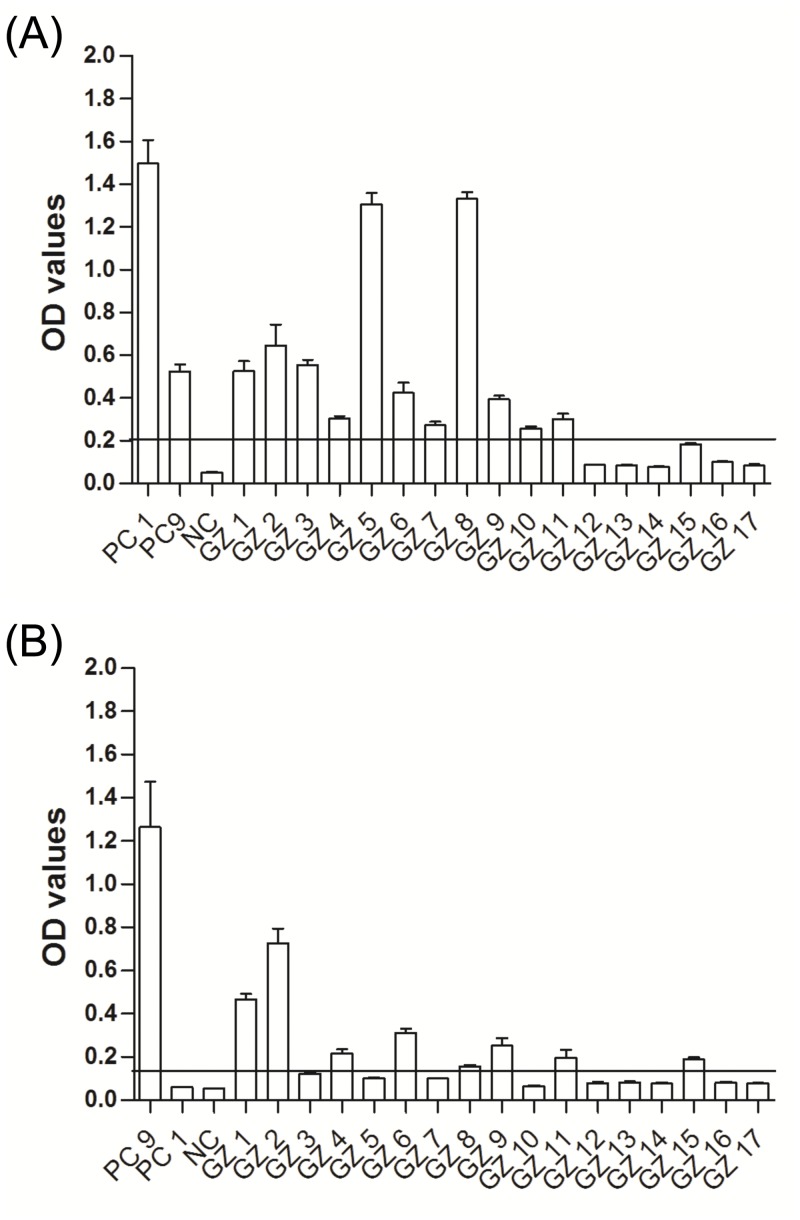
EHV antibodies in captive Grevy’s zebra. OD values of antibody responses tested by peptide-based ELISA in captive Grevy’s zebra using EHV-1_E (A) and EHV-9_G (B) peptides. Black line: cutoff above which samples are considered positive. PC1 = EHV-1 positive control (serum collected at day 21 from experimentally infected horse), PC9 = EHV-9 positive control (serum collected from an experimentally infected rabbit), NC = negative control (umbilical cord blood serum), GZ = Grevy’s zebra.

**Fig 5:**
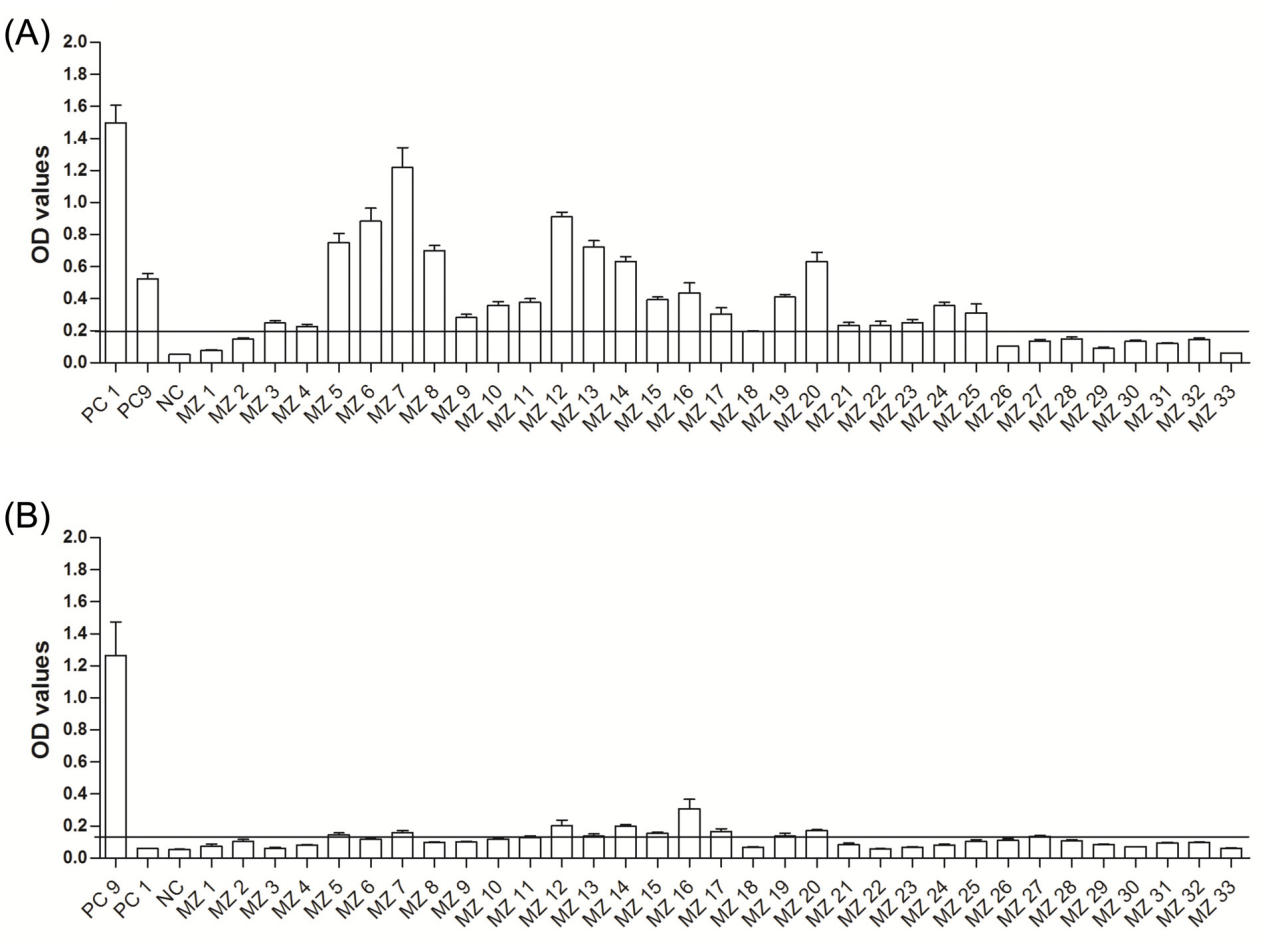
EHV antibodies in captive Hartmann's mountain zebra. OD values of antibody responses tested by peptide-based ELISA in captive Hartmann's mountain zebra using EHV-1_E (A) and EHV-9_G (B) peptides. Black line: cutoff above which samples are considered positive. PC1 = EHV-1 positive control (serum collected at day 21 from experimentally infected horse), PC9 = EHV-9 positive control (serum collected from an experimentally infected rabbit), NC = negative control (umbilical cord blood serum), MZ = Hartmann's mountain zebra.

All sera were tested by ELISA using EHV-1_E and EHV-9_G specific peptides (Table 1). In captive plains zebras, 22 and 10 serum samples tested positive for EHV-1 and EHV- 9 antibodies, respectively (n = 39; Fig 3A and 3B), 6 samples were classified as questionable for EHV-1, and one sample was questionable for EHV-9 antibodies. In wild plains zebras, 33 serum samples tested positive for EHV-1 exposure and 14 serum samples were positive for EHV-9 antibodies. The OD values in captive plains zebra were generally lower than those found in wild plains zebras (n = 41; Fig 3C and 3D). In captive Grevy’s zebra, fewer (11 and 8 serum samples, respectively, were positive for EHV-1 and EHV-9 (n = 17, Fig 4A and 4B). In captive Hartman mountain zebra 22 and 8 samples were positive for EHV-1 and EHV-9 antibodies, respectively (n = 33, Fig 5A and 5B). The data showed that the prevalence of EHV-1, but not EHV-9, antibodies in wild plains zebras was significantly higher than that in captive plains zebras (P = 0.02; Fisher’s exact test). There was no significant difference in EHV-1 (P = 0.6; Fisher’s exact test) and EHV-9 (P = 0.2; Fisher’s exact test) prevalence between the three tested captive zebra species.

**Fig 6:**
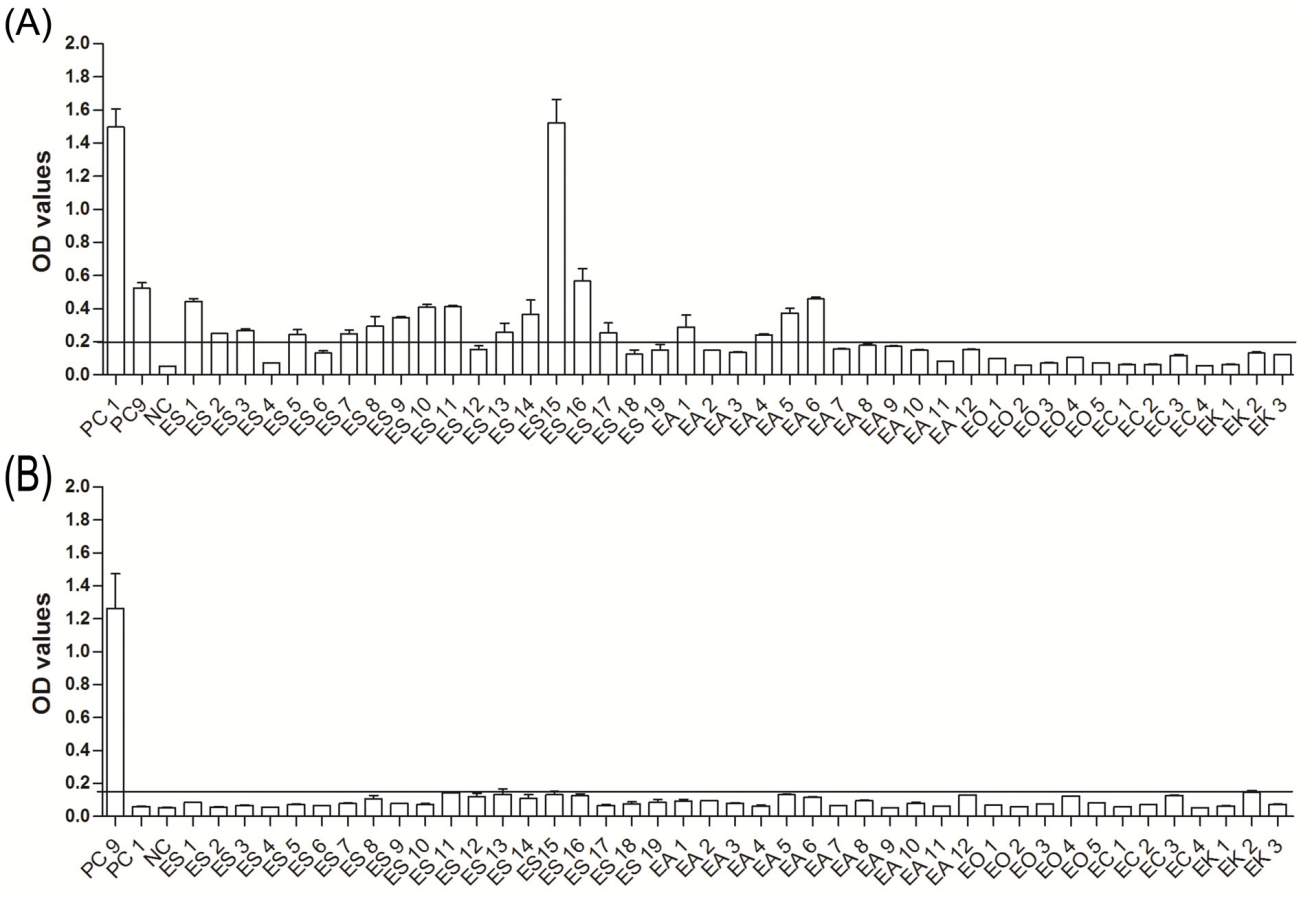
EHV antibodies in other captive equid species. OD values of antibody responses tested by peptide-based ELISA in other captive equid species using EHV-1_E (A) and EHV-9_G (B) peptides. Black line: cutoff above which samples are considered positive. PC1 = EHV-1 positive control (serum collected at day 21 from experimentally infected horse), PC9 = EHV-9 positive control (serum collected from an experimentally infected rabbit), NC = negative control (umbilical cord blood serum), ES = *Equus africanus somalicus* (Somali wild ass), EA = *Equus africanus asinus* (donkey), EO = *Equus hemionus onager* (onager), EC = *Equus ferus caballus* (pony), EK = *Equus hemionus kiang* (kiang).

Similar to the SNT results, onagers, ponies, and kiangs were negative for both EHV-1 and EHV-9 antibodies as detected with the ELISAs. Four donkeys tested positive (n = 12) for EHV-1 antibodies. In Somali wild asses, 14 samples were positive for EHV-1 antibodies and one was positive for EHV-9 antibodies (n = 19, Fig 6A and 6B). The prevalence of EHV-1 antibodies in the tested plains zebra (wild or captive), captive Hartmann’s zebra, and captive Somali wild ass sera was significantly higher than that of EHV-9 (P< 0.0001; Fisher’s exact test).

**Fig 7:**
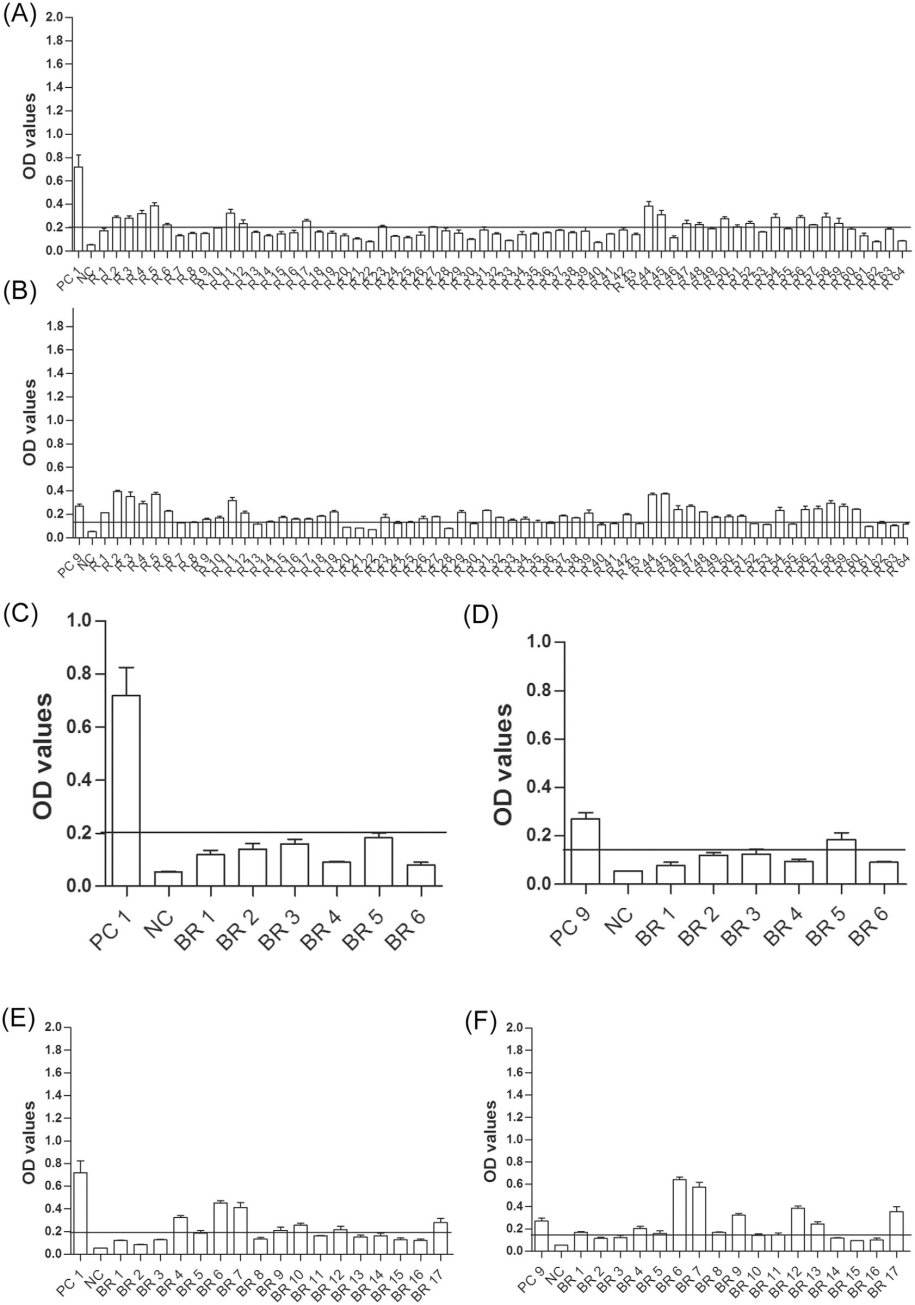
Prevalence of EHV-1 and EHV-9-antibodies in captive and wild rhinoceroses. OD values of antibody responses tested by peptide-based ELISA in captive white (A-B), black (C-D) and wild black (E-F) rhinoceroses using EHV-1_E (A, C, E) and EHV-9_G (B, D, F) peptides. Black line: cutoff above which samples are considered positive. PC = positive control (PZ27^w^) for EHV-1 and (PZ34^w^) for EHV-9, NC = negative control (umbilical cord blood serum), R = white rhino, BR = black rhino.

Out of 64 captive white rhinoceros sera, 20 serum samples tested positive for EHV-1 exposure and 42 tested positive for EHV-9 antibodies (Fig 7A and 7B). One of 6 tested captive black rhinoceroses’ serum samples was positive for EHV-9 antibodies (Fig 7C and 7D). In wild African black rhinoceros, 7 serum samples tested positive for EHV-1 exposure and 12 were positive for EHV-9 (n = 17, Fig 7E and 7F). The number of white rhinoceroses with exposure to EHV-9 was significantly higher than that with exposure to EHV-1 (P<0.05; Fisher’s exact test). Although the prevalence of EHV-9 antibodies was higher than that of EHV-1 in black rhinoceros (either wild or captive), we could not detect a significance difference after statistical analysis, which may be due to the small sample size. Moreover, the prevalence of the detected EHV-9 antibodies was significantly higher in the complete tested rhinoceros population than the zebra population, in both captive and wild animals, (P<0.05; Fisher’s exact test).

**Fig 8:**
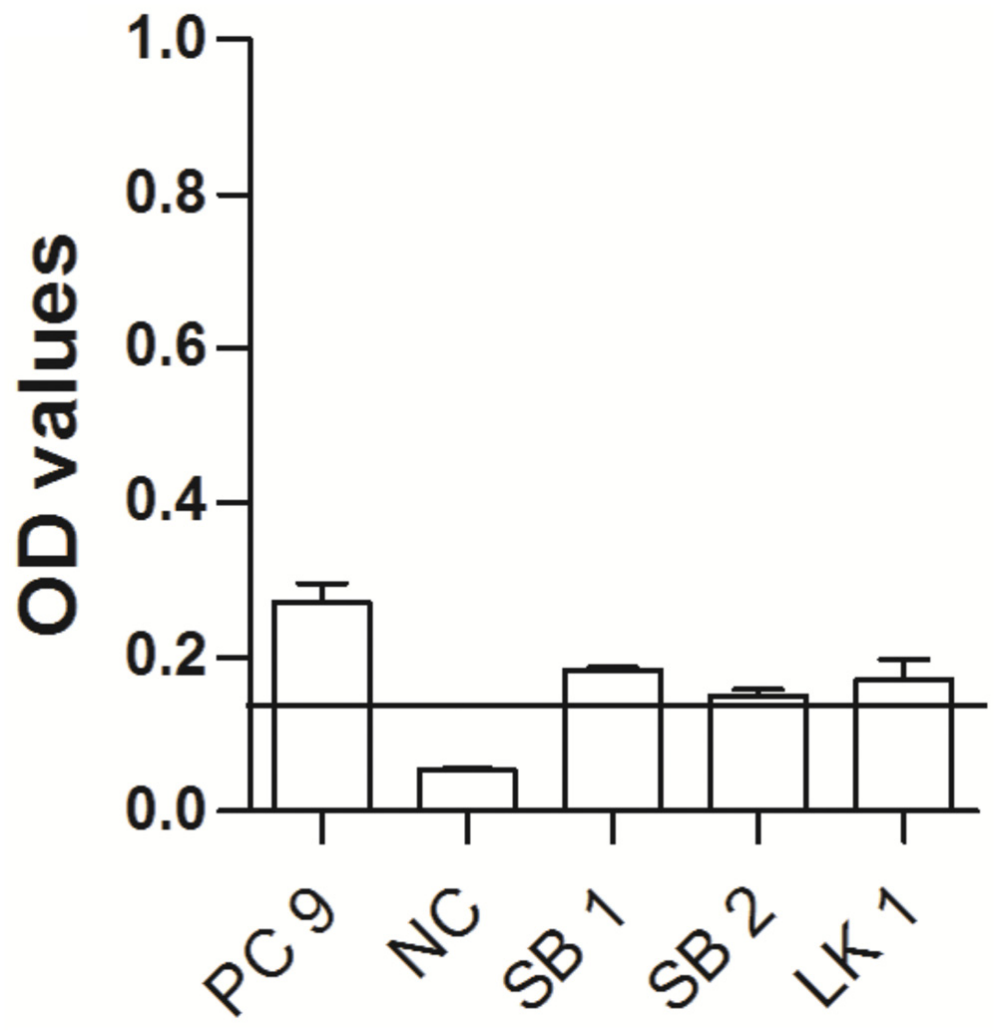
EHV antibodies in captive and wild antelopes. OD values of antibody responses tested by peptide-based ELISA in captive and wild antelope using EHV-9_G peptide. Black line: cutoff above which samples are considered positive. PC = positive control, PZ34^w^, NC = negative control (umbilical cord blood serum), SB = springbok, LK = lesser kudu.

The only EHV-9-positive captive antelope serum sample tested positive by SNT was confirmed as positive by ELISA. In addition, two free-ranging springboks (*Antidorcas marsupialis*) were weakly positive for EHV-9 antibodies but that was only evident in the ELISA (Fig 8).

None of the other animal families (*Giraffidae*, *Cervidae*, *Hippopotamidae*, *Cercopithecidae*, *Elephantidae*) tested positive for antibodies to either virus. We were unable to test for EHV-1 and EHV-9 antibodies in carnivores by ELISA (*Ursidae*, *Felidae*, *Canidae*, *Hyaenidae)* due to the high cross-reactivity of the protein A/G secondary antibody. The numbers of positive serum samples for each species are listed in details in Table 3.

**Table 3:** Equine herpesvirus-antibody positive rates in captive and wildlife sera using peptide-based ELISA.

		Number (%) of positive results	
*Family*/ animal	Total	EHV-1	EHV-9
Captive sera			
*Equidae*			
Plains zebra	39	22 (56.4%)	10 (25.6%)
Grevy's zebra	17	11 (64.7%)	8 (47%)
Hartmann's mountain zebra	33	22 (66.6%)	8 (24.2%)
Donkey	12	4 (33.3%)	0 (0.0%)
Somali wild ass	19	14 (73.6%)	1 (5.2%)
*Rhinocerotidae*			
White rhinoceros	64	20 (31.2%)	42 (65.6%)
Black rhinoceros	6	0	1 (16.6%)
*Bovidae*	37	0	1 (2.7%)
Wild sera			
*Equidae*			
Plains zebra	41	33 (80.4%)	14 (34.1%)
*Rhinocerotidae*			
Black rhinoceros	17	7 (41.1%)	12 (70.5%)
*Bovidae*	42	0 (0.0%)	2 (4.7%)

### Molecular finding

Out of 17 wild African black rhinoceros samples, 3 blood samples were available in addition to serum. DNA was extracted and a nested PCR amplification of a partial sequence of the DNA-dependent-DNA polymerase gene was applied [20, 32]. A 250 bp fragment was amplified from one sample. Sanger sequencing revealed a sequence with 94% homology to the novel gammaherpesviruses sequence described from Damara’s zebra (*E*. *burchellii antiquorum*), Somali wild ass (*E*. *asinus somalicus*), and eastern kiang (*E*. *kiang holdereri*) [12] (data not shown).

## Discussion

None of the animals that tested positive for antibodies to either virus exhibited clinical symptoms indicative of herpesvirus infection. This is characteristic of natural viral host species and may be suggestive of co-adaptation of species that are natural conspecifics of equid reservoir animals. Serological analysis was undertaken because latency, the hallmark of herpesvirus infection, would probably underestimate the prevalence based on presence of viral antigen or nucleic acid. Furthermore, serological assays can be used for detection of possible reservoir hosts as described previously [33]. EHV-1 and EHV-9 share high nucleotide sequence similarity, the overall difference of their complete genome sequences being 9% [7]. As a result of this high degree of similarity, it is difficult to distinguish EHV-1 from EHV-9 serologically. SNT is known to be a sensitive and robust test, but may not accurately discriminate between similar viruses [34]. The limitations are well known for discrimination of EHV-1 and EHV-4, which share much less antigenic similarity compared to the EHV-1 and EHV-9 pair. Nonetheless, it is impossible to distinguish between EHV-1 and EHV-4 by SNT [24]. The epidemiological data of equine herpesviruses in zoo and wildlife is very limited and based on SNT [3, 21]. As an illustration of the problems associated with exclusive reliance on SNT, we found that 26 of 59 equid sera, which tested positive for EHV-9 by SNT, were negative by ELISA, results indicating that the detected serum neutralizing EHV-9 antibodies were a result of cross-reactivity with EHV-1. In contrast, peptide-ELISA has been successfully applied as a specific and sensitive serological test for detection of EHV-1 and EHV-4 seroprevalence [24]. A recent study also demonstrated that ELISA was more sensitive than SNT in detection of EHV-1 antibodies in milk [35]. ELISA tests, particularly when using strain specific peptides, are more discriminating and sensitive than SNT.

While the EHV-9_G peptide was highly specific and could accurately discriminate the EHV-9 infected animals, the EHV-1_E peptide cross reacted with EHV-9 positive samples. With all EHV-1-positive but EHV-9-negative serum samples, OD values considered positive were obtained with the EHV-1_E peptide, while OD values with the EHV-9_G peptide were negative. Many of the serum samples testing positive for EHV-9 were also positive for EHV-1. Whether this represents cross reaction or co-infection in the given serum samples is unclear. However, the EHV-1_E peptides allowed the exclusion of EHV-4 infection [24]. While this does present some limitations in determining the specific exposure status of the animals examined, the results provide a much higher resolution determination of infection history, particularly for EHV-9, than was previously possible for such genetically similar viral strains.

Sampling was undertaken with two goals in mind. The first was to sample as many zebras as possible, both captive and wild to determine the prevalence of EHV-1 and EHV-9 in the presumptive host for both viruses. The second goal was to sample African con-specifics of zebras as broadly as possible to determine the range of species potentially infected rather than focusing on any one species. Peptide-based ELISA demonstrated that the prevalence of EHV-1 antibodies in equids (wild or captive plains zebra, captive Hartmann’s zebra, and captive Somali wild ass) was significantly higher than that of EHV-9. However, due to the cross reactivity of EHV-9 positive antibodies with the EHV-1_E peptide, further study will be needed to confirm the results obtained or refine the discriminatory power of the ELISA. These results strongly suggest that these equid species are a natural and definitive host for EHV-1. There was no significant difference in EHV-1 and EHV-9 prevalence between the three tested zebra species. The results were not influenced by zebra species, thus all zebras exhibited very similar prevalence. While wild Hartmann’s zebra and Somali wild ass sera were not available for this study, the lack of significant difference between wild and captive plains zebra and any plain zebra to the other equids suggests a similar prevalence could be expected in the wild for these species. EHV-1 and EHV-9 infections were reported previously in onager and pony [36, 37]; however, we could not detect antibodies for any of the viruses in these species, which might be due to the low sample size. In contrast, both EHV-1- and EHV-9-specific antibodies were detected in Somali wild asses. EHV-1 antibodies have been detected in Somali wild asses [18], however this is the first report of EHV-9 antibodies in this species. Recombination between EHV-1 and EHV-9 has been observed in sequences isolated from polar bear and Asian rhinoceros [19, 20]. The co-occurrence of these viruses in equids suggests that closely related equid alphaherpesviruses have natural opportunities to recombine and help explain the origin of recombinant isolates.

Using the developed peptide ELISA, an unexpectedly high prevalence of EHV-9 antibodies among captive and free-living African rhinoceros species was observed, the inverse of the observed prevalence in zebras, which exhibited higher EHV-1 antibody prevalence. However, EHV-1 antibodies were detected in larger number of rhinoceroses when compared to a previous study that relied exclusively on the immunofluorescence assay illustrating the higher sensitivity and specificity of the peptide based ELISA developed here [5]. The unexpectedly high prevalence of EHV-9 antibodies in rhinoceroses (particularly, captive white rhinoceros) suggests they are susceptible to EHV-9 infection and may serve as a natural and possibly definitive host or reservoir. The question of the natural reservoir and definitive host is particularly important for EHV-9 as the source of the many fatal infections of ungulates under natural conditions was not identified. Both EHV-1 and EHV-9 have been involved in fatal encephalitis cases in captive polar bears without proximity to equids [12, 19]. Rhinoceroses, which were not considered a potential source of infection, may have been involved in these unexplained transmission events. Similarly, a recent Asian rhinoceros fatality as a consequence of EHV-1 infection was suspected from a zebra source but may have derived from African rhinoceros [20]. Mammals in many African ecosystems congregate at water sources during periods of seasonal water shortage, which may explain transmission of viruses among perissodactyls and non-perissodactyls. For example, EHV-9 antibodies were detected in one captive lesser kudu and two wild springbok by SNT and/or ELISA. The low prevalence of EHV-9-positive antelopes as well as the previous fatal EHV-9 infection [7] supports the hypothesis that these species are accidental hosts of the virus. In addition to the serological evidence, the detection of zebra isolated equine gammaherpesviral DNA in one wild black rhinoceros blood sample illustrates that transmission of EHVs does occur within African ecosystems. The general lower OD values observed for zebras and rhinoceros in captivity likely represents lower exposure, re-exposure and viral reactivation in captivity similar to the observe loss of pathogens in mice after generations of captivity [38].

Carnivores can be infected with EHV-1 and/ or EHV-9 either experimentally as in dogs and cats causing neurotropic encephalitis and death [39, 40] or naturally in captive polar bears which died after displaying severe nervous manifestations [12, 19]. Neither EHV-1 nor EHV-9 was detected in any carnivore sample in the current study by SNT. However, ELISA could not be applied due to non-specific cross reaction with protein A/G. Nonetheless, our findings suggest that carnivores are less frequently infected by EHV or fail to seroconvert and based on experimental infection, may be more likely to exhibit neurological symptoms when infection does occur. African carnivores, many of which prey on or scavenge equids may have evolved resistance to EHV as a result of the high risk of exposure to infection. Polar bears and non-African carnivores would not be expected to have evolved such resistance which may explain the relatively frequent observed fatal disease in species that are not naturally sympatric with African Perissodactyls.

Taken together, we propose that EHV-1 and EHV-9 have evolved a broad host range among African mammals including distantly related perissodactyls. The results presented here show that different families including *Equidae*, *Rhinocerotidae* and *Bovidae*, respond with robust antibody responses to EHV-1 and EHV-9 exposure. The high prevalence in the *Rhinoceroteridae* in particular, suggests that they may be a natural host and/or reservoir for EHV-9. Further study is needed to determine the role of these animals in EHV epidemiology in both captivity and the wild.

## Supporting Information

S1 Fig Optimization of protein G-peroxidase. Ten negative and 10 positive zebra sera were tested with EHV-1_E (A) peptide and EHV-9_G (B) peptide. Black line: cutoff above which samples are considered positive. ^c^ = Captive serum sample, ^w^ = wild serum sample.
(EPS)

S1 Table Antibodies titers of 27 zebra sera used to calculate the negative cutoff value using SNT. PC = positive control (positive horse serum) for EHV-1 and (rabbit serum) for EHV-9, NC = negative control (fetal calf serum).
(DOCX)
