# Supplementary material for: Comprehensive Serology Based on a Peptide ELISA to Assess the Prevalence of Closely Related Equine Herpesviruses in Zoo and Wild Animals
Source: PLoS One. 2015 Sep 17;10(9):e0138370. doi: 10.1371/journal.pone.0138370 (PMC4574707; doi:10.1371/journal.pone.0138370)
Supplement: S1 Table — PC = positive control (positive horse serum) for EHV-1 and (rabbit serum) for EHV-9, NC = negative control (fetal calf serum). (DOCX) [file pone.0138370.s002.docx]

**S1 Table. SNT results of 27 zebra sera used to calculate the negative cutoff value.**

| Sample ID | Describtion | EHV-1 titer | EHV-9 titer |
| --- | --- | --- | --- |
| NC | Horse serum | <1:4 | <1:4 |
| PC |  | 1:32 | 1:64 |
| PZ29 | plains zebra | <1:4 | <1:4 |
| PZ30 | plains zebra | <1:4 | <1:4 |
| PZ31 | plains zebra | <1:4 | <1:4 |
| PZ32 | plains zebra | <1:4 | <1:4 |
| PZ33 | plains zebra | <1:4 | <1:4 |
| PZ34 | plains zebra | <1:4 | <1:4 |
| PZ35 | plains zebra | <1:4 | <1:4 |
| PZ36 | plains zebra | <1:4 | <1:4 |
| PZ37 | plains zebra | <1:4 | <1:4 |
| PZ38 | plains zebra | <1:4 | <1:4 |
| PZ39 | plains zebra | <1:4 | <1:4 |
| PZ41 | plains zebra | <1:4 | <1:4 |
| GZ12 | Grevy’s zebra | <1:4 | <1:4 |
| GZ13 | Grevy’s zebra | <1:4 | <1:4 |
| GZ14 | Grevy’s zebra | <1:4 | <1:4 |
| GZ15 | Grevy’s zebra | <1:4 | <1:4 |
| GZ16 | Grevy’s zebra | <1:4 | <1:4 |
| GZ17 | Grevy’s zebra | <1:4 | <1:4 |
| MZ25 | Hartmann’s mountain zebra | <1:4 | <1:4 |
| MZ26 | Hartmann’s mountain zebra | <1:4 | <1:4 |
| MZ27 | Hartmann’s mountain zebra | <1:4 | <1:4 |
| MZ28 | Hartmann’s mountain zebra | <1:4 | <1:4 |
| MZ29 | Hartmann’s mountain zebra | <1:4 | <1:4 |
| MZ30 | Hartmann’s mountain zebra | <1:4 | <1:4 |
| MZ31 | Hartmann’s mountain zebra | <1:4 | <1:4 |
| MZ32 | Hartmann’s mountain zebra | <1:4 | <1:4 |
| MZ33 | Hartmann’s mountain zebra | <1:4 | <1:4 |
